# Efficient Adsorption and Extraction of Glutathione S-Transferases with Glutathione-Functionalized Graphene Oxide–Polyhedral Oligomeric Silsesquioxane Composite

**DOI:** 10.3390/molecules28010340

**Published:** 2023-01-01

**Authors:** Jingqi Sun, Limin Jia, Xuwei Chen

**Affiliations:** Department of Chemistry, College of Sciences, Northeastern University, Box 332, Shenyang 110819, China

**Keywords:** octa-aminopropyl caged polyhedral oligomeric silsesquioxane (OA–POSS), graphene oxide (GO), glutathione S-transferases (GST), adsorption, solid-phase extraction

## Abstract

Glutathione S-transferases (GSTs) are important type-II detoxification enzymes that protect DNA and proteins from damage and are often used as protein tags for the expression of fusion proteins. In the present work, octa-aminopropyl caged polyhedral oligomeric silsesquioxane (OA–POSS) was prepared via acid-catalyzed hydrolysis of 3-aminopropyltriethoxysilane and polymerized on the surface of graphene oxide (GO) through an amidation reaction. Glutathione (GSH) was then modified to GO–POSS through a Michael addition reaction to obtain a GSH-functionalized GO–POSS composite (GPG). The structure and characteristics of the as-prepared GPG composite were characterized using scanning electron microscopy (SEM), Fourier transform infrared spectroscopy (FT-IR), thermogravity analysis, and surface charge analysis. The specific binding interactions between glutathione and GST gave GPG favorable adsorption selectivity towards GST, and other proteins did not affect GST adsorption. The adsorption behavior of GST on the GPG composite conformed to the Langmuir isotherm model, and the adsorption capacity of GST was high up to 364.94 mg g^−1^ under optimal conditions. The GPG-based solid-phase adsorption process was applied to the extraction of GST from a crude enzyme solution of pig liver, and high-purity GST was obtained via SDS-PAGE identification.

## 1. Introduction

Glutathione S-transferases (GSTs), a class of supergene family enzymes, are important type-II detoxifying enzymes in living organisms. GSTs are widely distributed in mammals, plants, and microorganisms [1,2,3], protecting DNA and proteins from damage mainly by catalyzing the binding of the sulfhydryl group of glutathione (GSH) to electrophilic substances [4]. Previous research determined that there are seven main cytoplasmic GSTs distributed in the human body with similar structures and partially overlapping functional regions [5]. Human cytoplasmic GSTs usually consist of a dimer of two homologous or heterologous subunits with a molecular weight of approximately 25.5 kDa [6,7,8,9]. The primary structure of GST contains two functional structural domains in each subunit, the C-terminal domain and the N-terminal domain, which are responsible for substrate-specific recognition and catalytic reactions [10,11].

Due to their involvement in the detoxification processes of many compounds, GSTs are usually used as biomarkers for a wide range of environmental assays [12,13]. In the medical field, GSTs are often considered as indicators of certain diseases such as lung cancer and other tumors [14,15,16]. In genetic engineering research, GST is used as a protein tag to construct fusion proteins due to the high efficiency of the GST fusion protein expression system and the ease of purifying the expressed products [17,18]. Glutathione (GSH) is a commonly adopted ligand in the isolation and purification of GSTs and GST-tagged proteins. Agarose-based GSH affinity materials are widely used commercial chromatography media for the purification of GSTs or GST-tagged proteins due to their easy derivatization and good biocompatibility [19]. Today, the design and development of cost-effective GSH affinity media have been attracting increasing attention [20,21,22], and GSH has been successfully modified on different substrates, including polystyrene (PS) [19], ultrafine fibers [22], magnetic nanoparticles [23,24,25], hollow silica [26], and hydrogel [27], to reduce costs and improve the efficiency of GST extraction.

Polyhedral oligomeric silsesquioxane (POSS) is a common organic–inorganic hybrid material that can form random, trapezoidal, and caged structures. As a representative POSS, octa-aminopropyl caged POSS (OA–POSS) has eight primary amino groups and a well-defined cubic octameric silica cage. Amino groups, in the form of chloride salts, can act as efficient conjugation sites for polymerization [28,29,30] and surface modification [30,31,32]. Due to its superior water-solubility and abundant reactive sites, OA–POSS has been used extensively as a fascinating candidate for constructing biocompatible polymer networks, and POSS-based composites have gained great popularity in various fields such as biology, chemistry, and medicine [33,34,35,36,37,38].

In the present study, OA–POSS was immobilized on the GO surface via an amidation reaction to obtain GO–POSS loaded with a large content of amino groups. Then, glutathione (GSH) was covalently modified on the GO–POSS surface via a Michael addition reaction to produce a GSH-modified GO–POSS composite (GPG). GPG was able to recognize and adsorb GST efficiently and specifically due to the enzyme–substrate interactions between GSH and GST. The practicability of the composite in the selective extraction of GST has been well demonstrated, providing an efficient approach for bio-separation.

## 2. Results and Discussion

### 2.1. Preparation and Characterization of GPG Composite

**Scheme 1:**
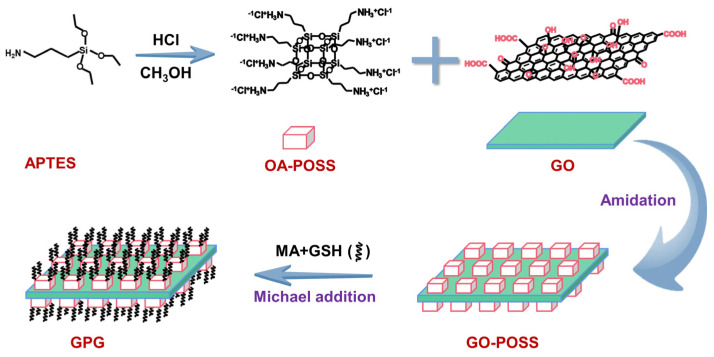
Preparation of the GPG composite.

Scheme 1 illustrates the preparation route for the GPG composite. OA–POSS was first obtained via the hydrolytic condensation reaction of APTES in an acidic methanol solution with concentrated HCl as the catalyst. Due to the excess hydrochloric acid in the reaction system, the terminal aminopropyl group of the produced OA–POSS was presented as ammonium chloride salt. Then, OA–POSS was covalently grafted onto the GO surface through an amidation reaction between the aminopropyl group of OA–POSS and the oxygen-containing groups (e.g., epoxy and carboxyl groups) of GO to obtain the GO–POSS composite. Thereafter, maleic anhydride was modified onto the vertices of GO–POSS via an amidation reaction, and glutathione was finally modified onto GO–POSS through a Michael addition reaction between the double bond of maleic anhydride and the sulfhydryl group of glutathione to give the final GPG composite product.

**Figure 1:**
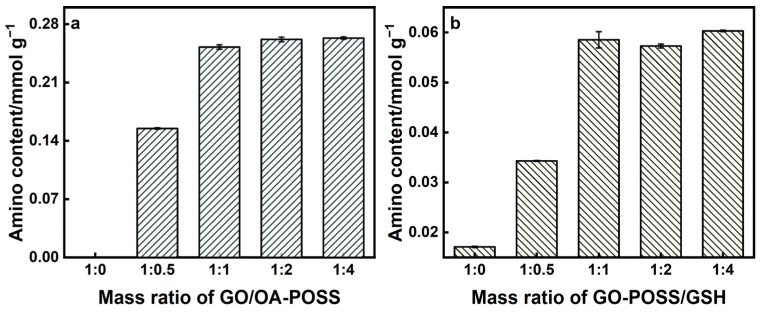
(**a**) Amino group contents on GO–POSS obtained with different GO/OA–POSS mass ratios. (**b**) Amino group contents on GPG composite obtained with different GO–POSS/GSH mass ratios.

To provide an efficient site for protein adsorption, the influence of the GO/OA–POSS and GO–POSS/GSH mass ratio on material preparation were investigated by quantifying the amino group content on the material’s surface via the fluorescence amine method. As shown in Figure 1a, the content of amino groups on GO–POSS first increased with the introduced OA–POSS amount, and then remained relatively stable when the OA–POSS amount further increased, suggesting that the GO surfaces were fully coated by OA–POSS under a GO/OA–POSS mass ratio of 1:1. A similar trend was observed for the amino group content on the surface of the GPG composite when the GO–POSS/GSH mass ratio changed from 1:0 to 1:4 (Figure 1b), indicating that the surface of GO–POSS was fully occupied by GSH when the GO–POSS/GSH mass ratio was higher than 1:1. Therefore, a GO/OA–POSS and GO–POSS/GSH mass ratio of 1:1 was adopted for GPG preparation, and the amino group on the final GPG composite makes the obtained GPG a hydrophilic Lewis base.

**Figure 2:**
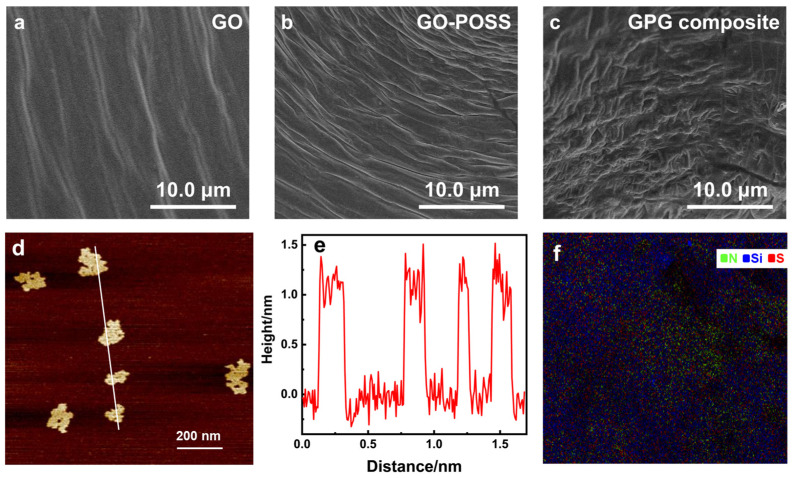
SEM images of (**a**) GO, (**b**) GO–POSS, and (**c**) GPG composite. (**d**) AFM image and (**e**) height profile of GO-POSS. (**f**) EDS mapping of GPG composite.

The scanning electron micrograph (SEM) images demonstrated that the as-prepared GO was a silk wave-like sheet with some undulating folds (Figure 2a), and the specific surface deduced from nitrogen adsorption–desorption measurement was 48.145 m^2^ g^−1^. It was obvious that the polymerization of OA–POSS on the surface changed the GO morphology greatly; the material surface became rougher with deep folds (Figure 2b).

Compared to GO and GO–POSS, more irregular folds were observed on the GPG composite (Figure 2c), indicating the successful modification of abundant GSH on the GO–POSS surface. Atomic force microscopy indicates that the thickness of GO-POSS sheet was ~1.31 nm (Figure 2d,e). The thickness of POSS layer is thus deduced to be ~0.52 nm, which is consistent to the size of the POSS cage (0.5 nm) [39], suggesting OA-POSS is monolayer polymerized on GO surface. EDS mapping result reveals that there were N, Si and S elements existed in GPG composite (Figure 2f), and these elements were evenly distributed.

**Figure 3:**
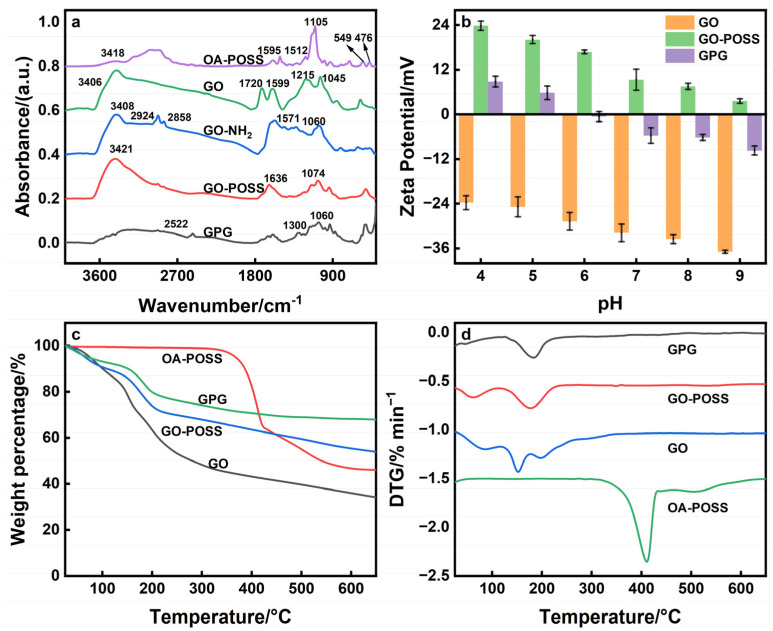
(**a**) FTIR spectra of OA–POSS, GO, GO-NH_2_, GO–POSS, and GPG composite. (**b**) Zeta potentials of GO, GO–POSS, and GPG composite. (**c**) TGA curves of GO, OA–POSS, GO–POSS, and GPG composite. (**d**) DTG curves of GO, OA–POSS, GO–POSS, and GPG composite.

The FTIR spectra of OA–POSS, GO, GO-NH_2_, GO–POSS, and GPG composite are shown in Figure 3a. In the FTIR spectrum of OA–POSS, the peaks centered at 3418 cm^−1^ and 1595 cm^−1^ were the characteristic stretching vibrational peaks of -NH_3_^+^ on the primary amine of POSS, and the three characteristic peaks located at 1105, 549, and 476 cm^−1^ were the stretching and formation vibrational peaks of the cubic inorganic silica oxygen cage. For GO, the broad peak at 3406 cm^−1^ was the stretching vibration peak of -OH, and the C=O (carboxyl), C-O-C (epoxy), and C-O (hydroxyl) stretching vibration peaks were located at 1720, 1215, and 1045 cm^−1^, respectively. In addition, the bands at 1599 cm^−1^ were the skeletal vibrations of graphite and the C-O-H vibrational absorption of carboxy/phenolic hydroxyls. The GO-NH_2_ modified with ethylenediamine showed peaks of 2924 cm^−1^ and 2858 cm^−1^, which were attributed to N-H (amine) and C-H (methylene) stretching vibrations. Peaks at 1571 cm^−1^ and 1060 cm^−1^ were attributed to the C=O and C-N stretching vibrations of the amide bonds. These indicated the successful modification of the amine (-NH_2_) on the GO surface. As for GO-POSS, the peaks corresponding to -NH-C(O)-stretching and Si-O-Si stretching vibrations appeared at 1636 cm^−1^ and 1074 cm^−1^, indicating that POSS was covalently bonded onto GO surface. The GPG composite showed new peaks at 2552 cm^−1^ and 1300 cm^−1^ for sulfhydryl and cyclic anhydride stretching vibration absorption, respectively, indicating the successful modification of GSH on GO-POSS.

Figure 3b illustrates the surface zeta potentials for the GO, GO–POSS, and GPG composite. In the pH range of 4–9, the presence of oxygen-containing groups (e.g., carboxyl and hydroxyl groups) on the surface resulted in a negative zeta potential for GO. GO–POSS exhibited a positive zeta potential, which was produced by the abundant amino groups on the surface. The GPG composite was positively charged at a pH lower than 6 and negatively charged when the pH was higher than 6, possibly because the isoelectric point (pI) of glutathione is 5.93. It is positively charged when pH < pI and negatively charged when pH > pI. The potential changes of the three materials indicated the successful preparation of the GO–POSS and GPG composite.

Figure 3c,d show the thermogravimetric analysis (TGA) and differential thermal gravity (DTG) curves of the GO, OA–POSS, GO–POSS, and GPG composite. GO was less thermally stable and exhibited obvious mass loss below 250 °C caused by the evaporation of water (<120 °C) and the decomposition of unstable oxygen-containing groups (carboxyl, epoxy and hydroxyl groups) on the surface (120~250 °C). The thermal decomposition of OA–POSS occurred mainly in temperature ranges of 320–430 and 430–560 °C. The mass loss in the first stage was mainly due to the thermal decomposition of the terminal aminopropyl of OA–POSS, and the mass loss in the second stage was ascribed to the thermal decomposition of inorganic cubic siloxane cage. GO–POSS exhibited better thermal stability than GO, which was attributed to the formation of thermally stable amide bonds between OA–POSS and the oxygen-containing groups of GO. The total mass loss of GPG composite at 600 °C was smaller than that of GO–POSS, indicating that the thermal stability of GPG was further enhanced by GSH modification.

### 2.2. Protein Adsorption Behaviors on GPG Composite

**Figure 4:**
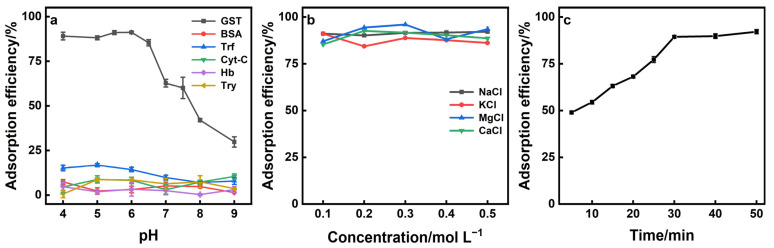
(**a**) Adsorption efficiencies of GST, BSA, Trf, Cyt-C, Hb, and Try onto the GPG composite at pH 4–9. GST solution: 1.0 mL and 100 mg L^−1^; adsorption time: 30 min; mass of adsorbent: 1.0 mg. (**b**) Dependence of the adsorption efficiency of GST at pH 6.5 on the variation of ionic strength within a range of 0.1–0.5 mol L^−1^ NaCl, KCl, MgCl_2,_ and CaCl_2_. Protein solution: 1.0 mL and 100 mg L^−1^; adsorption time: 30 min; mass of adsorbent: 1.0 mg. (**c**) Adsorption efficiency of GST onto the GPG composite under different adsorption times. Protein solution: 1.0 mL and 100 mg L^−1^; mass of adsorbent: 1.0 mg.

Several proteins common in organisms including bovine serum albumin (BSA, pI = 4.7), transferrin (Trf, pI = 5.9), cytochrome C (Cyt-C, pI = 10.7), hemoglobin (Hb, pI = 7.1), and trypsin (Try, pI = 10.1) were selected as models to study the protein adsorption behaviors on the GPG composite. As illustrated in Figure 4a, the adsorption of BSA, Trf, Cyt-C, Hb, and Try on the GPG composite in the pH range of 4–9 was low, as the retention of these protein species on the GPG surface was only driven by physical adsorption. On the other hand, favorable adsorption of GST was achieved in acidic circumstances, and an obvious decrease in GST adsorption was observed when pH became higher than 6.5. The reason for this result may be that the enzyme–substrate-specific interaction between GPG and GST was the main driving force for GST adsorption, resulting in a high adsorption efficiency with a low pH range. The isoelectric point (pI) of GST is 6.5 [40], and when pH > pI, GST became negatively charged. Thus, the electrostatic repulsion between the protein and the material led to a decrease in adsorption efficiency. Therefore, a PBS buffer of pH 6.5 was adopted for sample preparation in the present study. Figure 4b shows the effect of time on GST adsorption. The results showed that the adsorption efficiency first increased with time. When the time became longer than 30 min, no obvious change in adsorption efficacy was observed. Thus, an adsorption time of 30 min was used.

The influence of ionic strength on GST adsorption was investigated under pH 6.5 by adding a series of NaCl, KCl, MgCl_2,_ and CaCl_2_ (0.1–0.5 mol L^−1^) into the protein sample. As shown in Figure 4c, an increase of ionic strength had almost no influence on GST adsorption. This result indicated that electrostatic interactions are not the main driving force for the adsorption of GST onto the GPG composite, and the adsorption process can tolerate high salinity. It is also worth noting that the ionic strength equivalent to complex biological samples has no negative effect on GST adsorption, suggesting that the GPG composite is suitable for the extraction of GST from biological samples.

### 2.3. Thermodynamic Analysis of GST Adsorption by GPG Composite

**Figure 5:**
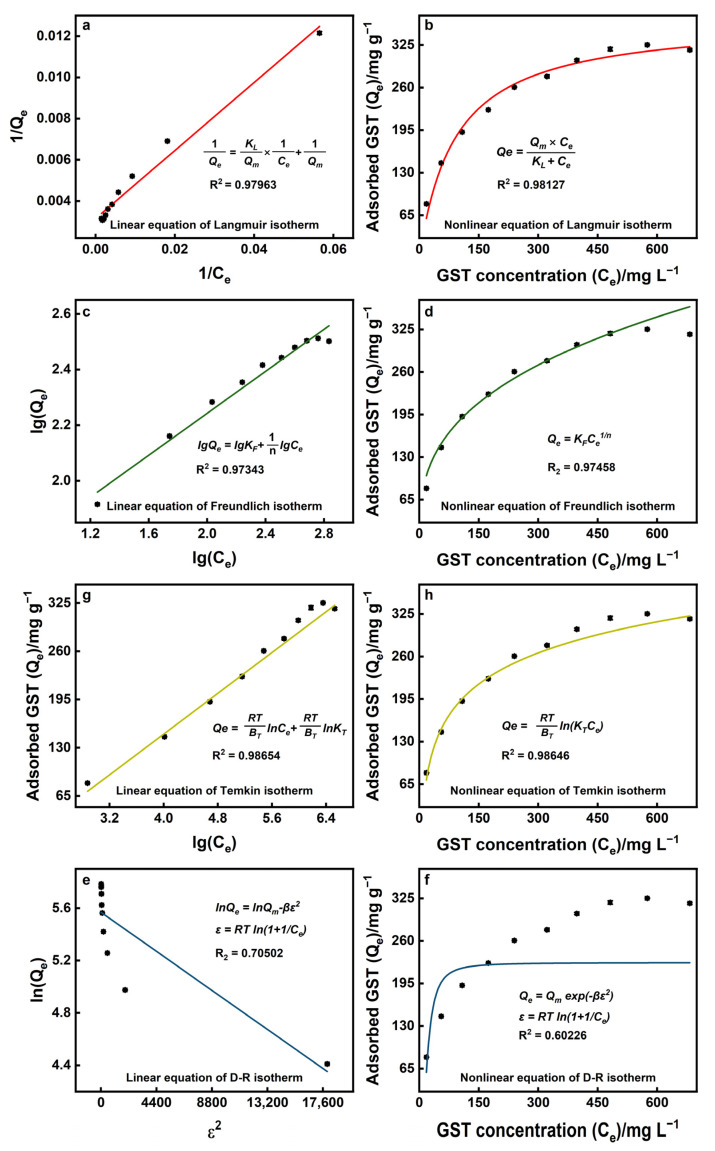
(**a**) The linear fit results of Langmuir isotherm model. (**b**) The nonlinear fit results of Langmuir isotherm model. (**c**) The linear fit results of Freundlich isotherm model. (**d**) The nonlinear fit results of Freundlich isotherm model. (**e**) The linear fit results of Temkin isotherm model. (**f**) The nonlinear fit results of Temkin isotherm model. (**g**) The linear fit results of Dubinin–Radushkevich isotherm model. (**h**) The nonlinear fit results of Dubinin–Radushkevich isotherm model. GST solutions: 100–1000 mg L^−1^ and 1.0 mL; mass of the adsorbent: 1.0 mg; adsorption time: 30 min.

The adsorption capacity of GPG toward GST was evaluated by studying the adsorption behaviors of a series of GST samples with concentrations ranging from 100 to 1000 mg L^−1^ at pH 6.5. The experimental results were fitted with Langmuir, Freundlich, Temkin and Dubinin–Radushkevich (D–R) isotherm models, respectively, and the results were illustrated in Figure 5.

The GST adsorption isotherm fit well with the Langmuir isotherm model, as given in Equation (1):

(1) $$Q_{e}=\frac{Q_{m}\times C_{e}}{K_{L}+C_{e}}$$

where *Q_m_* denotes the maximum adsorption capacity of GPG toward GST, *C_e_* represents the GST equilibrium concentration, *Q_e_* is the adsorbed content of GST by GPG, and *K_L_* represents the Langmuir constant. The *Q_m_* of GST onto GPG composite was deduced to be 364.94 mg g^−1^ by fitting the experimental data into the above equation (Figure 5a,b).

Table 1 summarizes the performance of reported materials for GST adsorption and separation. It can be seen that the adsorption capacity of GPG to GST was much higher than that of the reported materials. The reason for this result might lie in the fact that GO nanosheets offered a large specific surface area for the polymerization of OA–POSS, which provided abundant sites for GSH modification. The large amount of GSH thus contributed to the superior GST adsorption capacity of the GPG composite.

The fitted results obtained from Temkin isotherm model (Equation (2)) exhibited a higher correlation coefficient than Freundlich isotherm model (Equation (3)), thus it is presumed that the adsorption of GST on GPG composite is chemisorption (Figure 5c–f).

(2) $$Q_{e}=\frac{RT}{B_{T}}\ln\left(K_{T}C_{e}\right)$$

(3) $$Q_{e}=K_{F}C_{e}{}^{\frac{1}{n}}$$

*R* is the universal gas constant (8.314 J mol^−1^ K^−1^); *T* is the temperature (K); *K_T_* is the binding constant which corresponds to the maximum binding energy (L mg^−1^); *B_T_* is associated with adsorption heat (J mol^−1^); *K_F_* is Freundlich constant related to coefficient (mg g^−1^); *n* is the function of favorability of the system (g L^−1^).

In contrast, the R^2^ of the fitted results for D-R isotherm model (Equation (4)) is low, suggesting that GST is not adsorbed in the pore channel of the material (Figure 5g,h).

(4) $$Q_{e}=Q_{m}\exp\left(-\beta \varepsilon^{2}\right)$$

*β* is a constant corresponding to adsorption energy (mol^2^ kJ^−2^); ε is adsorption potential, expressed as RT ln(1 + 1/C_e_) [41].

The fitted parameters of the four sorption isotherm models are given in Table 2.

### 2.4. Kinetic Analysis of GST Adsorption by GPG Composite

To further investigate the adsorption process, the data were fitted using three kinetic models namely pseudo first order, pseudo second order and intraparticle diffusion models.

Pseudo first order kinetic model, pseudo second order, intraparticle diffusion kinetic model are expressed by the following Equations (5)–(7):

(5) $$Q_{t}=Q_{e}\left(1-exp^{-k_{1}t}\right)$$

(6) $$Q_{t}=\frac{K_{2}Q_{e}{}^{2}t}{1+K_{2}Q_{e}t}$$

(7) $$Q_{t}=kt^{0.5}+c$$

where *k*_1_ is the pseudo first order rate constant (min^−1^), *k*_2_ is the pseudo second order rate constant (g mg ^−1^ min^−1^), *k* is the intra particle diffusion coefficient (mg g^−1^ min^−0.5^); *C* is the boundary layer thickness [41,42]. The fitted parameters of the three adsorption kinetic models are given in Table 3.

**Figure 6:**
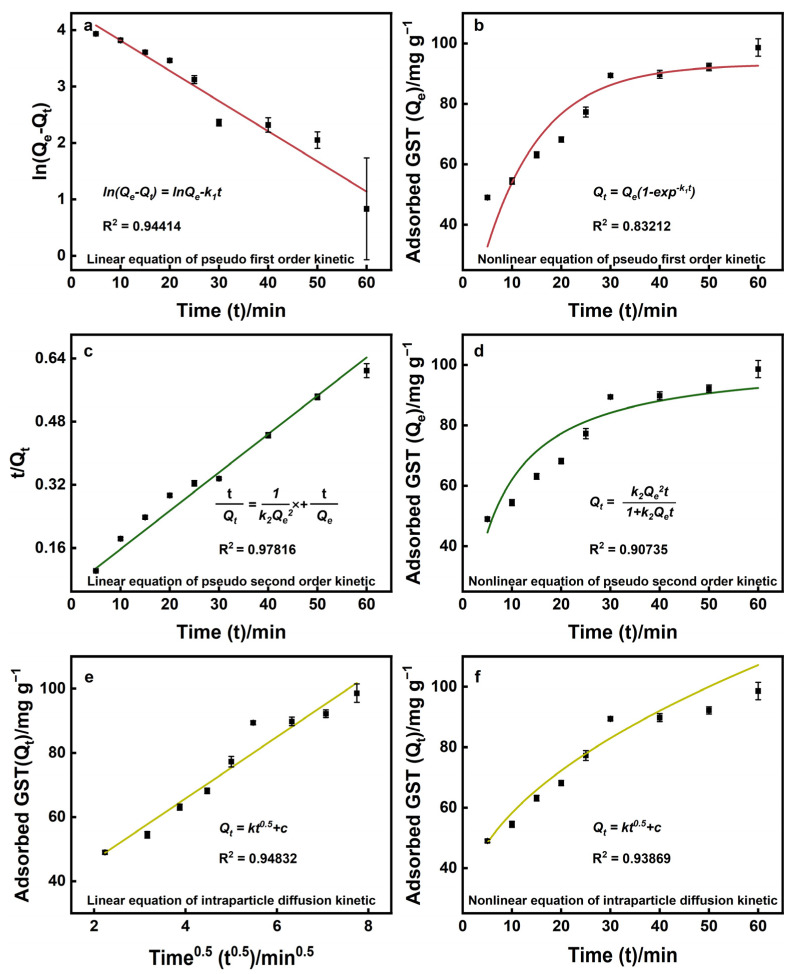
(**a**) The linear fit result of pseudo first order kinetic model. (**b**) The nonlinear fit result of pseudo first order kinetic model. (**c**) The linear fit result of pseudo second order kinetic model. (**d**) The nonlinear fit result of pseudo second order kinetic model. (**e**) The linear fit result of intraparticle diffusion kinetic model. (**f**) The nonlinear fit result of intraparticle diffusion kinetic model. GST solution: 1.0 mL, 100 mg L^−1^; Mass of adsorbent: 1.0 mg; pH 6.5.

Comparison on the pseudo first order and pseudo first order kinetics revealed that the adsorption process was more consistent with quasi-secondary kinetics, indicating that the adsorption of GST on the GPG composite was more inclined to chemisorption (Figure 6a–d). Based on the results of the intraparticle diffusion fitting, it is clear that intraparticle diffusion dominates the adsorption process. The linear line does not pass through the origin probably due to the fact that the adsorption process is controlled by a multi-step mechanism (Figure 6e,f).

### 2.5. Recovery of GST from GPG Composite

**Figure 7:**
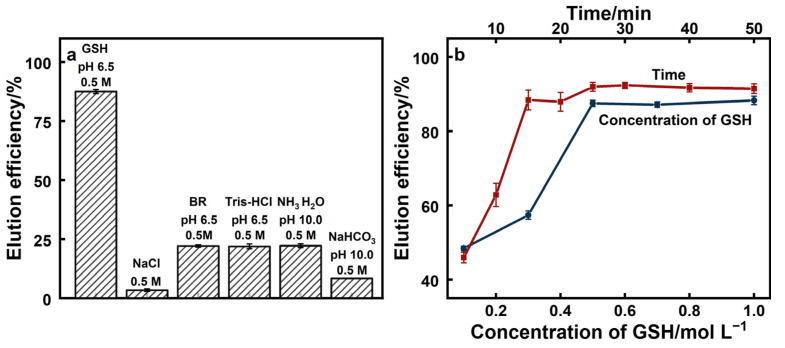
(**a**) The elution efficiencies of adsorbed GST from GPG with different stripping reagents. GST solution: 1.0 mL and 100 mg L^−1^; adsorption time: 30 min; elution time: 20 min; mass of adsorbent: 1.0 mg. (**b**) The elution efficiencies of adsorbed GST from GPG under different GSH concentrations and elution times. GST solution: 1.0 mL and 100 mg·L^−1^; adsorption time: 30 min; mass of adsorbent: 1.0 mg.

For efficient recovery of GST from the GPG composite, several potential reagents, including GSH (pH 6.5, 0.5 mol L^−1^), NaCl (0.5 mol L^−1^), Tris-HCl (pH 6.5, 0.5 mol L^−1^), BR buffer (pH 6.5, 0.5 mol L^−1^), ammonia (pH 10.0, 0.5 mol L^−1^), and NaHCO_3_ (pH 10.0, 0.5 mol L^−1^), were used for GST elution. The results showed that GSH (pH 6.5, 0.5 mol L^−1^) offered the best elution efficiency (Figure 7a), which might benefit from the intense binding competition from free GSH to GST. 

Figure 7b shows the influence of the GSH concentration and elution time. It was found that GST could be effectively eluted from a GPG composite with a GSH concentration higher than 0.5 mol L^−1^ and an elution time longer than 15 min. To ensure efficient GST recovery, a GSH concentration of 0.5 mol L^−1^ and an elution time of 20 min were ultimately adopted.

### 2.6. Reusability and Stability of GPG Composite

**Figure 8:**
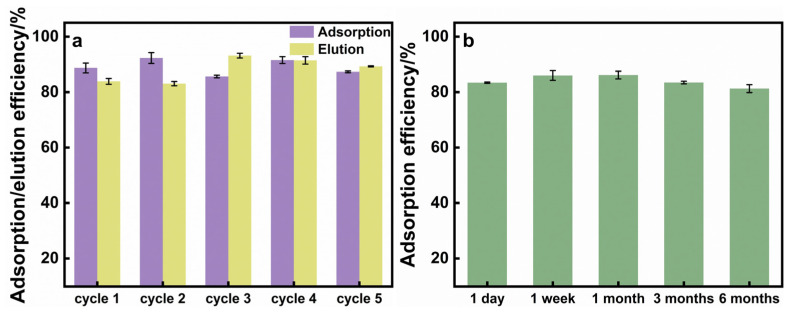
(**a**) The adsorption and elution efficiency of GST by GPG for 5 repeated adsorption–elution cycles. GST solution: 1.0 mL, 100 mg L^−1^; Adsorption time: 30 min; Elution time: 20 min; Mass of adsorbent: 1.0 mg. (**b**) Adsorption efficiency of GST onto GPG composite under different storage time. GST solution: 1.0 mL, 100 mg L^−1^; Adsorption time: 30 min; Elution time: 20 min; Mass of adsorbent: 1.0 mg.

Figure 8a shows the adsorption and elution efficiency of GST on a GPG composite for continuous adsorption–elution processes. After five repeated adsorption–elution cycles, no deterioration in adsorption and elution efficiency was observed, indicating that the GPG composite offers favorable reusability. Figure 8b illustrates the GST adsorption performance of GPG composites after different placement times. Ultimately, GPG still offered good GST adsorption performance after long-term placement. Additionally, an adsorption efficiency higher than 80% was achieved, even after six months of storage, indicating that the as-prepared GPG composite has good long-term stability.

### 2.7. Extraction of GST from Synthetic Sample and Pig Live

The practical applications of the GPG composite were evaluated by extracting GST from the synthetic sample and pig liver.

**Figure 9:**
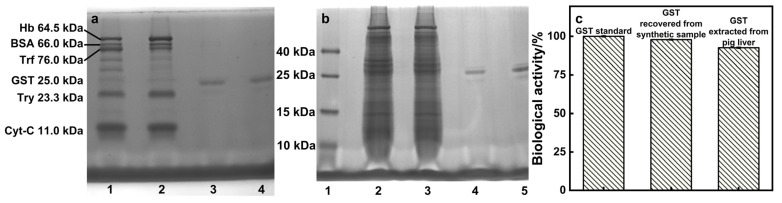
(**a**) SDS-PAGE assay results of the synthetic sample. Lane 1: synthetic sample; lane 2: synthetic sample after adsorption by the GPG composite; lane 3: solution recovered from the GPG composite; lane 4: GST standard (200 μg mL^−1^). (**b**) SDS-PAGE assay results of the pig liver sample. Lane 1: molecular weight standards (Marker in kDa); lane 2: 5-fold diluted pig liver sample without pretreatment; lane 3: 5-fold diluted pig liver sample after adsorption by the GPG composite; lane 4: GST-recovered solution from the GPG composite; lane 5: GST standard (200 μg mL^−1^). (**c**) Biological activity of GST after the adsorption–elution process.

The synthetic sample was composed of six protein species of different concentrations (500 μg mL^−1^ for BSA, Trf, Cyt-C, Hb, and Try, as well as 100 μg mL^−1^ for GST). After adsorption/elution by the GPG composite, the synthetic sample before/after adsorption and the eluate were collected and subjected to an SDS-PAGE assay. As shown in Figure 9a, the bands of each protein species (Trf, 76 kDa; BSA, 66 kDa; Hb, 64.5 kDa; GST, 25 kDa; Try, 23.3 kDa; Cyt-C, 11 kDa) were clearly observed in the synthetic sample (Lane 1). The GST band basically disappeared in the simulated sample lane after adsorption (Lane 2), and a clear band appeared in the eluate (Lane 3), which was at the same position as that in the GST standard, suggesting the excellent adsorption selectivity of the GPG composite to GST.

Figure 9b illustrates the SDS-PAGE results of the pig liver crude enzyme solution. The presence of multiple distinct bands in the range of 10–40 kDa in the crude enzyme solution indicates the complex protein composition of the pig liver (Lane 2). After adsorbing the GPG composite, these protein bands were still present in the supernatant, suggesting that these proteins were not adsorbed by GPG (Lane 3). In the eluate, a distinct band appeared only at 25 kDa (Lane 4), which was consistent with the standard GST band (Lane 5), while the other protein bands disappeared completely, indicating that the recovered GST was of high purity. These results demonstrate that GPG was able to achieve the selective extraction of high-purity GST from a complex biological matrix.

The activity of the recovered GST was determined via 1-chloro-2,4-dinitrobenzene (CDNB)-based colorimetry [43,44], and the results are shown in Figure 9c. After the adsorption and elution processes, the GST recovered from the synthetic sample retained 97.85% activity, and the GST extracted from the pig liver exhibited 92.65% bioactivity when compared to the GST standard, indicating that the GPG composite was of favorable biocompatibility and could be used to extract highly active GST from biological samples.

## 3. Materials and Methods

### 3.1. Chemicals and Reagents

Glutathione S-transferase (GST, G6511), Bovine serum albumin (BSA, A1933), transferrin (Trf, T8158), Cytochrome C (Cyt-C, G6511), Hemoglobin (Hb, G6511), Trypsin (Try, T8802), and 1-chloro-2,4-dinitrobenzene (C_6_H_3_ClN_2_O_4_, CDNB) were purchased from Sigma-Aldrich (St. Louis, MO, USA); Glutathione (GSH), graphite powder, maleic anhydride (MA), fluorescamine, N,N,N′,N′-tetramethylethylenediamine (TEMED), sodium dodecyl sulfate (SDS), acrylamide, N,N′-Methylenebis (acrylamide), brilliant blue G-250, brilliant blue R-250, potassium persulfate (K_2_S_2_O_8_), ammonium persulfate (APS), DL-dithiothreitol (DDT), and 3-aminopropyltriethoxysilane (APTES) were obtained from Aladdin (Shanghai, China). The protein marker and loading buffer were provided by Takara Biomedical Technology (Dalian, China). Phosphorus pentoxide (P_2_O_5_), sulfuric acid (H_2_SO_4_, 98%), hydrogen peroxide solution (H_2_O_2_, 28~30%), potassium permanganate (KMnO_4_), tris(hydroxymethyl)aminomethane (Tris), ethylenediamine tetraacetic acid (EDTA), ethanediamine (EDA), and other chemicals were acquired from Sinopharm Chemical Reagent (Shanghai, China) and were at least analytical reagents. Unless otherwise stated, all chemicals used were at least analytically pure, and 18 MΩ cm deionized water was used in all experiments. 

### 3.2. Preparation of OA–POSS

Octa-aminopropyl caged polyhedral oligomeric silsesquioxane (OA–POSS) was prepared using a typical acid-catalyzed hydrolysis reaction of APTES [45]. Briefly, 160 mL of anhydrous methanol was added into a 500 mL beaker. Then, 20 mL of APTES was slowly added, and the mixture was stirred until well mixed. Thereafter, 27 mL of concentrated hydrochloric acid (36.5%, 12 mol L^−1^) was slowly added drop-wise. The resulting mixture was placed on a magnetic stirrer and stirred for one week at room temperature. After centrifugation at 10,000 rpm for 10 min, the product was collected and washed with anhydrous methanol until the supernatant became neutral. Then, the product was dried in a vacuum oven at 60 °C for 12 h to obtain white powder OA–POSS.

### 3.3. Preparation of GO-NH*_2_*

GO was synthesized from natural graphite by adopting the Hummers method [1] with some modifications [46,47]. Firstly, graphite powder (30.0 g) was pre-treated by mixing with concentrated H_2_SO_4_ (30 mL), K_2_S_2_O_8_ (10.0 g), and P_2_O_5_ (105.0 g), followed by heating the mixture at 80 °C for 6 h under continuous stirring. Next, an aliquot volume of deionized water was added, and the product was filtered through a 1.2 μm cellulose membrane, washed by water until neutral, and dried at 60 °C under a vacuum overnight. The pre-oxidized graphite (20.0 g) was then mixed with concentrated H_2_SO_4_ (460 mL) in an ice water bath at 0 °C. Afterwards, 60 g potassium permanganate was added into the above dispersion with drastic mechanical stirring to keep the temperature below 20 °C. Thereafter, the mixture was stirred at 35 °C for 2 h followed by continuously stirring at room temperature for 14 days. The viscose brown product was then made to react with H_2_O_2_ (3%, *v*/*v*) to remove the excess KMnO_4_. After separation via centrifugation at 10,000 rpm for 10 min, the collected suspension was washed using an HCl solution (1:10 *v*/*v*) and deionized water successively until the impurities were completely stripped, and a neutral supernatant was obtained. Finally, graphene oxide was collected and dried under a vacuum at 60 °C for 36 h. 

GO-NH_2_ was produced through the amination of GO using ethylenediamine [48]. In total, 0.2 g of GO was dispersed into 20 mL of ethanol and sonicated for 30 min. Then, ethylenediamine (EDA) was added and stirred vigorously at room temperature for 12 h. The obtained GO-NH_2_ was dried in a water bath at 80 °C under continuous stirring until the ethanol evaporated completely from the suspension. Lastly, the product was dried under a vacuum at 60 °C for 12 h.

### 3.4. Preparation of GO–POSS

GO–POSS was prepared through an amidation reaction between the carboxyl group on the surface of the GO and the amino group of OA–POSS. In total, 0.01 g of OA–POSS was dispersed in 30 mL of methanol, and 0.1 g of GO was added into 50 mL of methanol and ultrasonically dispersed for 60 min. Then, the OA–POSS solution was mixed with the GO solution, and the resulting mixture was kept at 60 °C under magnetic stirring for 24 h to achieve a reaction. After the reaction, the solution was centrifuged at 10,000 rpm for 15 min, and the collected product was washed with deionized water to remove unreacted POSS. The final product was dried in an oven at 60 °C for 24 h.

### 3.5. Preparation of GPG Composite

Maleic anhydride was first attached onto the GO–POSS surface via an amidation reaction [49]. Then, 1.0 g of GO–POSS was dispersed into 50 mL of toluene solution, and maleic anhydride was added into the system and refluxed at 160 °C under an N_2_ atmosphere for 2 h. Next, the toluene solvent was removed via rotary evaporation, and the GO–POSS nanoparticles modified with maleic anhydride were washed with deionized water and dried in an oven at 60 °C for 24 h.

Glutathione (GSH) was then used to modify the GO–POSS via a Michael addition reaction to obtain the final GPG composite product. Next, 100 mg of maleic-anhydride-modified GO–POSS nanosheets was dispersed in a PBS solution (0.1 mol L^−1^, pH 6.0), 100 mg of glutathione was added into the suspension, and the resulting mixture was incubated in PBS solution for 48 h. After centrifugation to remove the supernatant, the product was washed with deionized water and dried under a vacuum at 60 °C for 12 h.

The GSH content on the GPG composite was determined via the fluorescamine method [50,51] by recording the fluorescence intensity at 478 nm with excitation at 392 nm.

### 3.6. Characterization of the GPG Composite

Scanning electron microscopic (SEM) images were collected on a SU8010 field-emission electron microscope with S-3 and a voltage of 5.0 KV (Hitachi, Japan). Thermogravimetric Analysis (TGA) was performed on a Thermogravimetric Analyzer TGA/DSC 1/1600 LF (Mettler Tolede Switzerland) with a temperature range of 25 to 800 °C. The photoluminescent spectra were recorded on an F-7000 fluorescence spectrophotometer (Hitachi, Japan). UV–vis absorption spectra were recorded on a U-3900 UV–Vis spectrophotometer (Hitachi, Japan). DYCZ-24DN and DYCZ-24EN electrophoresis cells (LiuYi Beijing) were used for protein electrophoresis, and gel images were obtained using a Tanon 4600SF gel imaging system (Tanon Shanghai). An NW10VF ultrapure water system was used to obtain analytically pure water. Fourier transform infrared (FT-IR) spectra were collected using a Vertex 70 spectrophotometer (Bruker, Germany) with KBr pellets in the range of 400 to 4000 cm^−1^.

### 3.7. Protein Adsorption with GPG Composite

In total, 50 μL of GPG suspension (20 mg mL^−1^) and 100 μL of protein (1 mg mL^−1^) were mixed with 850 μL PBS buffer (20 momol L^−1^, pH 6.5), and the mixture was oscillated for 30 min at room temperature to facilitate protein adsorption onto the GPG composite. Next, the supernatant was collected via centrifugation at 10,000 rpm for 5 min, and the concentration of residual protein content in the supernatant was quantified using Coomassie Brilliant Blue (Bradford method). 

To elute the adsorbed protein, the GPG composite and retained protein were mixed with 1 mL GSH (pH 6.5, 0.5 mol L^−1^), and the resulting solution was incubated for 20 min to promote stripping of the adsorbed protein from the adsorbent surface.

The equilibrium adsorption capacity of protein onto the GPG composite was obtained using a series of GST concentrations from 100 to 1000 mg L^−1^.

### 3.8. Extraction of GST from Pig Liver with GPG Composite

Fresh pig liver (10 g) was washed with water twice and cut into small pieces, mixed with 100 mL of 0.01 mol ^−1^ phosphate buffer (pH 7.4, containing 3 mol L^−1^ DTT, 1 mol L^−1^ EDTA and 1 mol·L^−1^ ammonium sulfate), and placed into a homogenizer to obtain a homogeneous suspension. The suspension was first centrifuged at low speed (4 °C, 3000 rpm) for 10 min and then at high speed (4 °C, 10,000 rpm) for 20 min. Finally, the supernatant was collected as the crude enzyme solution.

In total, 1 mg of GPG composite was added to 1 mL crude enzyme solution. The resulting mixture was shaken for 30 min for protein adsorption. After centrifugation at 10,000 rpm for 5 min, the solid phase was collected and washed with deionized water, and then 1 mL of GSH solution (pH 6.5, 0.5 mol L^−1^) was added and shaken for 20 min to recover the adsorbed protein.

For SDS-PAGE analysis, the supernatant, and recovered and initial solutions were mixed with a loading buffer and boiled for 5 min. An SDS-PAGE assay was performed using 12% polyacrylamide separation gel and 5% polyacrylamide stacking gel. The protein bands were visualized by staining with Coomassie brilliant blue R-250 (0.1%, *w*/*v*) followed by decolorizing with 0.5 mol L^−1^ KCl.

## 4. Conclusions

In summary, in this study, a GPG composite was obtained through the polymerization of OA–POSS on GO nanosheets and covalently attaching GSH on the surface of GO–POSS. The huge specific surface area of GO nanosheets provides abundant available sites for subsequent modifications, and the fabrication process was characterized by SEM, AFS, EDS, FTIR, TGA and surface charge analysis. These characterizations well demonstrated that OA–POSS was monolayer polymerized on GO surface, and high density GSH was functionalized on the final composite. The enzyme–substrate-specific interaction of GSH with GST enables the efficient and highly selective adsorption of GST on the GPG composite. The adsorption of GST on GPG composite fits well with Langmuir isotherm model, and other protein species commonly existed in real biological sample do not interfere GST adsorption. GPG composite exhibited a high adsorption capacity of up to 364.94 mg g^−1^ towards GST, which was much higher than that of reported materials. At the same time, the GPG composite owned favorable reusability and good long-term stability. The practicability of the GPG composite was demonstrated by the successful extraction of GST from real biological sample, i.e., pig liver crude enzyme solution. The GPG composite was demonstrated to be of high biocompatible and the biological activity of isolated GST was well-maintained, which is of great significance for further biological research. This work not only provide an efficient sorbent for GST extraction, but also demonstrates that the construction of POSS-based functional materials via the rational selection of substrate and ligands is an effective strategy to provide biocompatible object-oriented adsorbents and achieve the efficient and highly selective extraction of biological molecules.

## Figures and Tables

**Table 1:** GST adsorption capacity on different materials.

Adsorbent	Adsorption Capacity (mg g^−1^)	Ref.
PLLA-PCys-GSH/PLGA fibers	6.5	[22]
SPION@silica-GSH nanoparticles	6.84	[24]
MNP-GSH magnetic nanoparticles	60	[25]
GPG composite	364.94	This work

**Table 2:** Summary of isotherm parameters of GST adsorption on GPG.

T = 20 °C	Linear			Nonlinear		
Models	R^2^	Parameter 1	Parameter 2	R^2^	Parameter 1	Parameter 2
Langmuir	0.9796	Q*_m_* = 363.63 mg g^−1^	*K_L_* = 99.48 mg L^−1^	0.9813	*Q_m_* = 364.94 mg g^−1^	*K_L_* = 90.13 mg L^−1^
Freundlich	0.9734	*K_F_* = 30.79 mg g^−1^	*n* = 2.65	0.9746	*K_F_* = 37.50 mg g^−1^	*n* = 2.89
Temkin	0.9865	*K_T_* = 0.159 L mg^−1^	*B_T_* = 35.97 J mol^−1^	0.9865	*K_T_* = 0.145 L mg^−1^	*B_T_* = 35.97 J mol^−1^
D-R	0.7050	*Q_m_* = 216.87 mg g^−1^	*β* = 6.77 × 10^−5^ mol^2^ kJ^−2^	0.6023	*Q_m_* = 226.80 mg g^−1^	*β* = 7.49 × 10^−5^ mol^2^ kJ^−2^

**Table 3:** Summary of kinetics parameters of GST adsorption on GPG.

T = 20 °C	Linear			Nonlinear		
Models	R^2^	Parameter 1	Parameter 2	R^2^	Parameter 1	Parameter 2
Pseudo first order	0.9441	*Q_e_* = 77.58 mg g^−1^	*k*_1_ = 0.0536 min^−1^	0.8321	*Q_e_* = 93.07 mg g^−1^	*k*_1_ = 0.0867 min^−1^
pseudo second order	0.9782	*Q_e_* = 103.0 mg g^−1^	*k*_2_ = 0.0016 g mg^−1^ min^−1^	0.9074	*Q_e_* = 102.4 mg g^−1^	*k*_2_ = 0.0015 g mg^−1^ min^−1^
intraparticle diffusion	0.9483	*c* = 27.36 mg g^−1^	*k* = 9.6049 mg g^−1^ min^−0.5^	0.9387	*c* = 24.46 mg g^−1^	*k* = 10.68 mg g^−1^ min^−0.5^

## Data Availability

The data presented in this work are available in the article.
